# Blib is a multi-module simulation platform for genetics studies and intelligent breeding

**DOI:** 10.1038/s42003-022-04151-9

**Published:** 2022-11-03

**Authors:** Luyan Zhang, Huihui Li, Jiankang Wang

**Affiliations:** 1 National Key Facility for Crop Gene Resources and Genetic Improvement, and Institute of Crop Sciences, Chinese Academy of Agricultural Sciences (CAAS), Beijing, 100081 China; 2 National Nanfan Research Institute (Sanya), Chinese Academy of Agricultural Sciences (CAAS), Sanya, Hainan 572024 China

**Keywords:** Plant breeding, Agricultural genetics, Plant hybridization

## Abstract

Simulation is an efficient approach for the investigation of theoretical and applied issues in population and quantitative genetics, and animal and plant breeding. In this study, we report a multi-module simulation platform called Blib, that is able to handle more complicated genetic effects and models than existing tools. Two derived data types are first defined in Blib, one to hold the required information on genetic models, and the other one to represent the genetics and breeding populations. A number of subroutines are then developed to perform specific tasks. Four case studies are present as examples to show the applications of Blib, i.e., genetic drift of multiple alleles in randomly mating populations, joint effects of neutral mutation and genetic drift, comparison of mass versus family selection, and choice of testers in hybrid breeding. Blib together with its application modules, has great potential to benefit theoretical genetic studies and intelligent breeding by simulating and predicting outcomes in a large number of scenarios, and identifying the best optimum selection and crossing schemes.

## Abstract (web summary)

Blib handles complex genetic effects and simulates large amounts of scenarios to identify the most optimum selection and cross schemes for intelligent breeding.

## Introduction

Simplified assumptions are often made for reasons of tractability in many theoretical studies, however the real world is more complicated. Computer simulation provides an efficient tool that has been widely used in many scientific fields and disciplines, probably since computers were invented. Genetic studies and breeding applications allow us to investigate the implications of relaxing some of the assumptions made in population and quantitative genetics, and their effects on the activities of breeding programs^1^. On the other hand, simulation can generate a large amount of data that is impossible or difficult to obtain from the empirical experiments or theoretical considerations, which can be used to compare different selection methods, and validate the proposed theories or models. In addition, simulation can help perform pilot research, accelerate research and development, and transfer discoveries from the laboratory or theory to practice^2,3^. As an example, by using molecular data on parents and genomic prediction models, the segregating populations of virtual crosses can be generated and then compared, from which the most promising ones can be predicted before the crosses are actually made in the field^4^.

To use the simulation approach in genetic studies and breeding applications, the development of suitable software packages, platforms and/or modules is fundamental. QU-GENE is such a platform that was developed for the quantitative analysis of genetic models^5,6^. Based on the QU-GENE platform, several application modules have been developed, and then used to investigate various issues in breeding. As an example module of QU-GENE, QuLine was designed to simulate the breeding programs for deriving inbred (or pure) lines and has been used to compare different breeding methods^7–9^, predict cross performance using known gene information^10^, optimize marker assisted selection in the pyramiding of multiple genes^11^, and investigate the efficiency of a single backcrossing breeding strategy^12^. QuHybrid is another module designed to simulate breeding programs for selecting hybrids as the final products, and it has been used to calculate the probability of success of a biofortified breeding program for improving the provitamin A content in maize^13^. More recently, QuMARS was developed to simulate and optimize various recurrent selection strategies, including phenotypic selection, marker-assisted recurrent selection, and genomic selection (GS) for both short-term and long-term breeding procedures^14^.

Several other simulation platforms have been developed in past decades for various purposes. SimuPop provides a forward-time simulation environment, which may be used to manipulate genetic populations after many generations of random mating, and investigate the evolutionary mechanism of natural populations^15^. PedigreeSim simulates meiosis in both diploid and tetraploid species and can be used to generate pedigrees and cross populations^16^. Slim is a forward simulation tool designed to study the effects of linkage and selection and is capable of modeling the complex scenarios of demography and population substructure^17^. Forqs is a forward-in-time simulator of recombination, quantitative traits and selection designed to investigate haplotype patterns in scenarios where substantial evolutionary change has taken place^18^. By using progeny simulation and genomic prediction strategies, PopVar can predict the population mean, genetic variance, mean of superior progenies, and correlated responses of multiple traits^19^. AlphaSim is a package for simulating plant and animal breeding programs that is flexible in terms of historical population structure and diversity, pedigree structure, trait architecture and selection strategy^20^. Similar to QU-GENE application modules, ADAM-plant can simulate breeding programs for self-pollinated and cross-pollinated crop plants, and for the application of new technologies such as speed breeding and GS^21^.

The QU-GENE platform together with its application modules represents one of the most mature and widely used simulation tools in genetics and breeding. The platform can handle a wide range of genetic models including molecular markers as well as genes underlying phenotypes, genetic linkage, multiple alleles, additive, dominance, epistasis, and gene-by-environment interactions. However, it was originally designed without considering more complicated effects and/or genetic phenomena, such as mutations, cytoplasm effects, and fertilities of female and male gametes, needless to say the interactions between cytoplasm and nuclear genes. These phenomena or effects are important in evolutionary biology, population genetics and breeding applications, but cannot easily be imbedded into the existing platform and its application modules.

In this study, we report a multi-module genetic simulation platform called Blib, which has the potential for a much wider range of genetic studies and intelligent breeding. First, we introduce the technical issues for derived data types of the generalized genetic models and genetics/breeding populations, together with major subroutines in Blib. We then present four case studies as examples to show some of the applications of Blib in genetic studies and breeding.

## Results

### Case study I: Genetic drift of multiple alleles in randomly mating populations

One Blib application module called DRIFT was developed to simulate genetic drift in randomly mating populations. First, two subroutines, i.e., Blib_ReadGmodel and Blib_ReadPopulation, were called to assign the global variable Gmodel and one initial population from two external files, respectively. Then, the subroutine Blib_Cross1Population was repeatedly called to conduct random mating in one parental population and generate the progeny population for a given number of generations. The whole procedure was repeated for a given number of runs or replications. Statistical parameters associated with the population were calculated by calling subroutine Blib_CalcPstatistics and outputted to external files for each generation of random mating and each run. One locus with multiple alleles but without mutation was considered in the genetic model. Two population sizes (*N*) were set at 10 and 50. For each population size, four initial allele numbers were considered in the base populations, i.e., 2, 0.5 *N*, *N* and 2 *N*. The alleles had equal frequency in the base populations. The drift procedure was repeated for 100 generations of random mating and 100 simulation runs. For each generation, allelic frequencies in the 100 subpopulations (generated from the 100 simulation runs) were recorded.

The inbreeding coefficient in limited-size populations during random drift has been well studied in population genetics^22–24^. Starting from one base population, the theoretical inbreeding coefficient after *t* generations of random drift in populations with the size of *N* is given by Eq. (1). Assuming there is no inbreeding in the base population, i.e., *F*_0_ = 0, we have Eq. (2).

1 $${F}_{t}=1-\left(1-\frac{1}{2N}\right)^{t}(1-{F}_{0})$$

2 $${F}_{t}=1-\left(1-\frac{1}{2N}\right)^{t}$$

Equations (1) and (2) are derived under some idealized conditions, such as fixed population size, equal fertility of female and male individuals, equal fertility of female and male gametes, and no generation overlap^23^. Under the nonidealized conditions, Eqs. (1) and (2) are applicable when replacing *N* with the effective population size (i.e. *N*_*e*_). Assuming there are a number of subpopulations under genetic drift starting from one base population, the observed inbreeding coefficient, i.e., *F*_*Obs*_, can be estimated from the observed allelic frequencies in subpopulations, i.e.,

3 $${H}_{Obs}	=\frac{1}{m}\mathop{\sum }\limits_{j=1}^{m}\left(1-\mathop{\sum }\limits_{i=1}^{k}{p}_{ij}^{2}\right),\,{H}_{Exp}=1-\mathop{\sum }\limits_{i=1}^{k}{\bar{p}}_{i}^{2}, \\ {F}_{Obs}	=\frac{{H}_{Exp}-{H}_{Obs}}{{H}_{Exp}}$$

where *p*_*ij*_ is the observed frequency of the *i*^th^ allele (*i* = 1 to *k*, where *k* is the number of alleles) in the *j*^th^ subpopulation (*j* = 1 to *m*, where *m* is the number of subpopulations, or the number of simulation runs in this study), and ${\bar{p}}_{i}$ is the mean frequency of the *i*^th^ allele across the *m* subpopulations^23^.

**Fig. 1:**
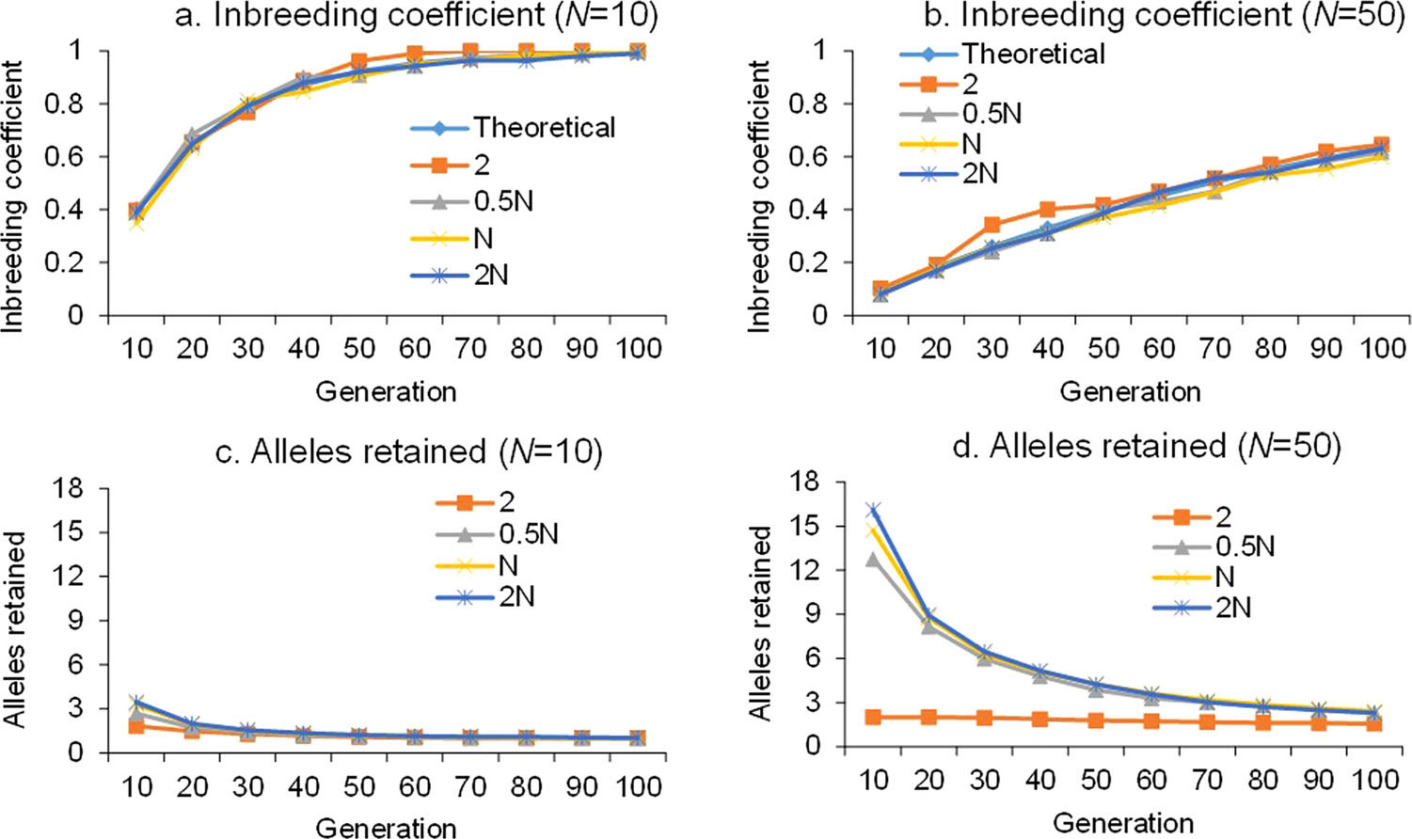
Theoretical and simulated inbreeding coefficients and the observed allele numbers after 100 generations of random drift for two population sizes (N) and four initial numbers of alleles. **a** Inbreeding coefficient when *N* is 10. **b** Inbreeding coefficient when *N* is 50. **c** Alleles retained when *N* is 10. **d** Alleles retained when *N* is 50.

Figure 1a, b show the change in the inbreeding coefficient during the 100 generations of random drift for two population sizes, 10 and 50, respectively, where the theoretical values were calculated by Eq. (2) and the observed values were calculated by Eq. (3). Obviously, the observed inbreeding coefficients are independent of the number of alleles (*k*) occurring in the base population, even though *k* is included in Eq. (3). The inbreeding coefficients estimated from Eq. (3) are highly consistent with their theoretical values, as given by Eq. (2). For a population size of 10, the inbreeding coefficient approaches one after 60-70 generations of random mating; for a population size of 50, the inbreeding coefficient approaches ~0.5 after 60–70 generations. The inbreeding coefficient is expected to approach one after infinite generations of random drift in limited-size populations. When the inbreeding coefficient becomes one, each subpopulation is fixed for one homozygous genotype, which is normally called the fixed or pure line. A simulation experiment based on the Blib application module DRIFT confirms the theory on inbreeding coefficients and its validity in the case of multiple alleles at one genetic locus.

Figure 1c, d show the change in allele number during the 100 generations of random drift for two population sizes. The observed number of alleles in one subpopulation was equal to the number of alleles with nonzero frequencies. Unlike the inbreeding coefficient, Fig. 1c, d indicate that the number of alleles retained in subpopulations depends on the number of alleles in base populations in earlier generations of random drift. The more alleles there are in the base population, the more alleles are retained, especially when the population size is large. It is expected that the allele number will also approach one after infinite generations of random drift in limited-size populations. The number of alleles located at one chromosomal locus is an important parameter for characterizing the genetic architecture of populations^22^. The change in allele number during random drift is difficult to study theoretically, but can be properly investigated through simulation.

### Case study II: Neutral mutations in randomly mating population with limited size

Neutral theory plays an important role in modern population genetics and evolution theories^24–28^. When mutation is included in the genetic model, the Blib application module as introduced and used in Case study I can also be used to simulate the simultaneous action of mutation and drift during random mating. Under mutation, the inbreeding coefficient between two succeeding generations is given in Eq. (4), where *N* is the population size, and *u* is the mutation rate^22,24^. According to neutral theory, an equilibrium can be reached after infinite generations of neutral mutation and genetic drift in limited-size populations. The theoretical inbreeding coefficient at equilibrium is shown in Eq. (5), which can be acquired from Eq. (4) by letting ${F}_{t}={F}_{t-1}$ and $1/{(1-u)}^{2}\approx 1+2u$. The number of alleles at mutation–drift equilibrium was investigated by Crow and Kimura^22^, Ewens^29^, and Karlin and McGregor^30^. Equation (6) is an approximate expression given by Ewens^29^, where $\theta =4Nu$.

4 $${F}_{t}=\left[\frac{1}{2N}+\left(1-\frac{1}{2N}\right){F}_{t-1}\right]{(1-u)}^{2}$$

5 $$\tilde{F}=\frac{1}{1+4Nu}$$

6 $$E(k)=\frac{\theta }{\theta }+\frac{\theta }{\theta +1}+\frac{\theta }{\theta +2}+\ldots +\frac{\theta }{\theta +n-1}$$

In practice, assuming there are a number of subpopulations under mutation and drift at generation *t*, the observed inbreeding coefficient can be estimated from the observed allelic frequencies in *m* subpopulations^24^, i.e.,

7 $${F}_{Obs}=\frac{1}{m}\mathop{\sum }\limits_{j=1}^{m}\mathop{\sum }\limits_{i=1}^{k}{p}_{ij}^{2}$$

where *p*_*ij*_ is the observed frequency of the *i*^th^ allele (*i* = 1 to *k*, where *k* is the number of alleles) in the *j*^th^ subpopulation (*j* = 1 to *m*, where *m* is the number of subpopulations, or the number of simulation runs in this study).

In the simulation using the Blib application module DRIFT, one locus with multiple alleles and mutations was considered in the genetic model. Two rates at the level of pairwise mutation were considered, i.e., 0.0001 and 0.0002. Two population sizes (*N*) were set at 10 and 50. For each population size, four initial allele numbers were considered in base populations, i.e., 2, 0.5 *N*, *N* and 2 *N*. All alleles had equal frequencies in the base populations. The mutation and drift procedure was repeated for 200 generations of random mating and 100 simulation runs. For each generation, allelic frequencies in the 100 subpopulations (generated from the 100 simulation runs) were recorded.

Table 1 shows the theoretical and observed inbreeding coefficients, together with the number of retained alleles in the population. Theoretical inbreeding coefficients at equilibrium were calculated by Eq. (5), and theoretical numbers of retained alleles after 200 generations were calculated by Eq. (6), where the total mutation rate was used. The total mutation rate listed in Table 1 is the pairwise mutation rate multiplied by *k*-1, where *k* is the number of alleles. Observed inbreeding coefficients after 200 generations were calculated by Eq. (7), and observed numbers of the retained alleles after 200 generations were acquired by counting the alleles with nonzero frequencies in subpopulations. The difference between the theoretical and observed values in Table 1 is marginal for both the inbreeding coefficient and the number of retained alleles. Even though neutral theory is built on the infinite-alleles model^24^, simulation results indicate that the theory on inbreeding coefficient and retained alleles works well under the scenario of a limited number of alleles at a genetic locus, even for the two-allele model (Table 1). Simulation using the Blib application module DRIFT provides solid evidence to demonstrate the wide application of neutral theory in population genetics.

**Table 1:** Theoretical inbreeding coefficient and number of retained alleles at equilibrium, and observed inbreeding coefficient and number of retained alleles after 200 generations of random drift and neutral mutation.

Scenario	Pairwise mutation rate	Population size (*N*)	Initial alleles	Total mutation rate (*u*)	Inbreeding coefficient		Final alleles	
					Theoretical	Observed	Theoretical	Observed
1	0.0001	10	2	0.0001	0.9960	0.9975	1.01	1.01
2	0.0001	10	0.5 *N*	0.0004	0.9843	0.9909	1.06	1.03
3	0.0001	10	*N*	0.0009	0.9653	0.9620	1.13	1.16
4	0.0001	10	2 *N*	0.0019	0.9294	0.9045	1.26	1.36
5	0.0001	50	2	0.0001	0.9804	0.9198	1.10	1.26
6	0.0001	50	0.5 *N*	0.0024	0.6757	0.6411	3.20	3.29
7	0.0001	50	*N*	0.0049	0.5051	0.5415	5.12	5.16
8	0.0001	50	2 *N*	0.0099	0.3356	0.3376	8.34	8.48
9	0.0002	10	2	0.0002	0.9921	0.9972	1.03	1.02
10	0.0002	10	0.5 *N*	0.0008	0.9690	0.9632	1.11	1.14
11	0.0002	10	*N*	0.0018	0.9328	0.9045	1.25	1.34
12	0.0002	10	2 *N*	0.0038	0.8681	0.8836	1.51	1.49
13	0.0002	50	2	0.0002	0.9615	0.9304	1.20	1.29
14	0.0002	50	0.5 *N*	0.0048	0.5102	0.4966	5.04	5.14
15	0.0002	50	*N*	0.0098	0.3378	0.3559	8.28	7.69
16	0.0002	50	2 *N*	0.0198	0.2016	0.2177	13.44	12.77

In fact, in Blib, mutation rates at any genetic locus are defined by one *k* by (*k*-1) matrix, where *k* is the number of alleles at the locus (Supplementary Fig. 1). For example, for one locus with three alleles, the first row of the matrix is mutation rates from allele 1 to alleles 2 and 3; the second row is mutation rates from allele 2 to alleles 1 and 3; and the third row is mutation rates from allele 3 to alleles 1 and 2. Therefore, nonuniform mutation rates can be defined in Blib, such as forward mutations having higher rates than reverse mutations or alleles at different loci having different mutation rates. It is anticipated that the effects of heterogeneous mutation rates on neutral theories, e.g. Equations (4) to (6), can be quantified as well by further simulation experiments using the Blib application module DRIFT.

### Case study III: Comparison of mass selection with family selection

One other Blib application module called PRS was developed to simulate the phenotypic recurrent selections that are typical in both animal and plant breeding. The selection in PRS can be based on phenotypic values at the individual level, or on phenotypic means of the progeny families. First, two subroutines Blib_ReadGmodel and Blib_ReadPopulation were called to assign the global variable Gmodel and one initial population from two external files, respectively. Then, the subroutine Blib_Cross1Population was called to conduct random mating in one parental population and generate a new progeny population. Blib_CalcPhenovalue was called to calculate phenotypic values for the progeny population. Blib_Select was called to conduct the selection. Finally, Blib_CalcPstatistics was called to calculate the statistical parameters associated with the population.

Four selection methods were considered in PRS, i.e., phenotypic or mass selection, which is based on the performance of individuals (PS); S1 family selection, which is based on the performance of one generation of selfed families (S1); S2 family selection, which is based on the performance of two generations of selfed families (S2); and half-sib selection, which is based on the performance of half-sib families (HS). Forty cycles of recurrent selection were conducted. Upward selection was conducted for 20 cycles, followed by 20 cycles of downward selection.

Two alleles were considered at each locus in the genetic model. Size of the initial population was set at 100. Models with three types of effects were simulated, i.e., pure additive effects (AD0), additive and dominant effects (AD1), and additive, dominant and epistatic effects (ADE). Ten independent genes were predefined to affect the trait of interest. For model ADE, five epistatic networks were considered. In each network, genes at two different loci interact with each other. Genotypic values at each individual locus or in each epistatic network are randomly assigned values between 0 and 1. Broad-sense heritability was set at 0.2 for each model. Mutation and no mutation were considered separately. When mutation was included, the rates of forward and reverse mutation were set at 0.02 and 0.01, respectively, for all genetic loci in consideration.

**Fig. 2:**
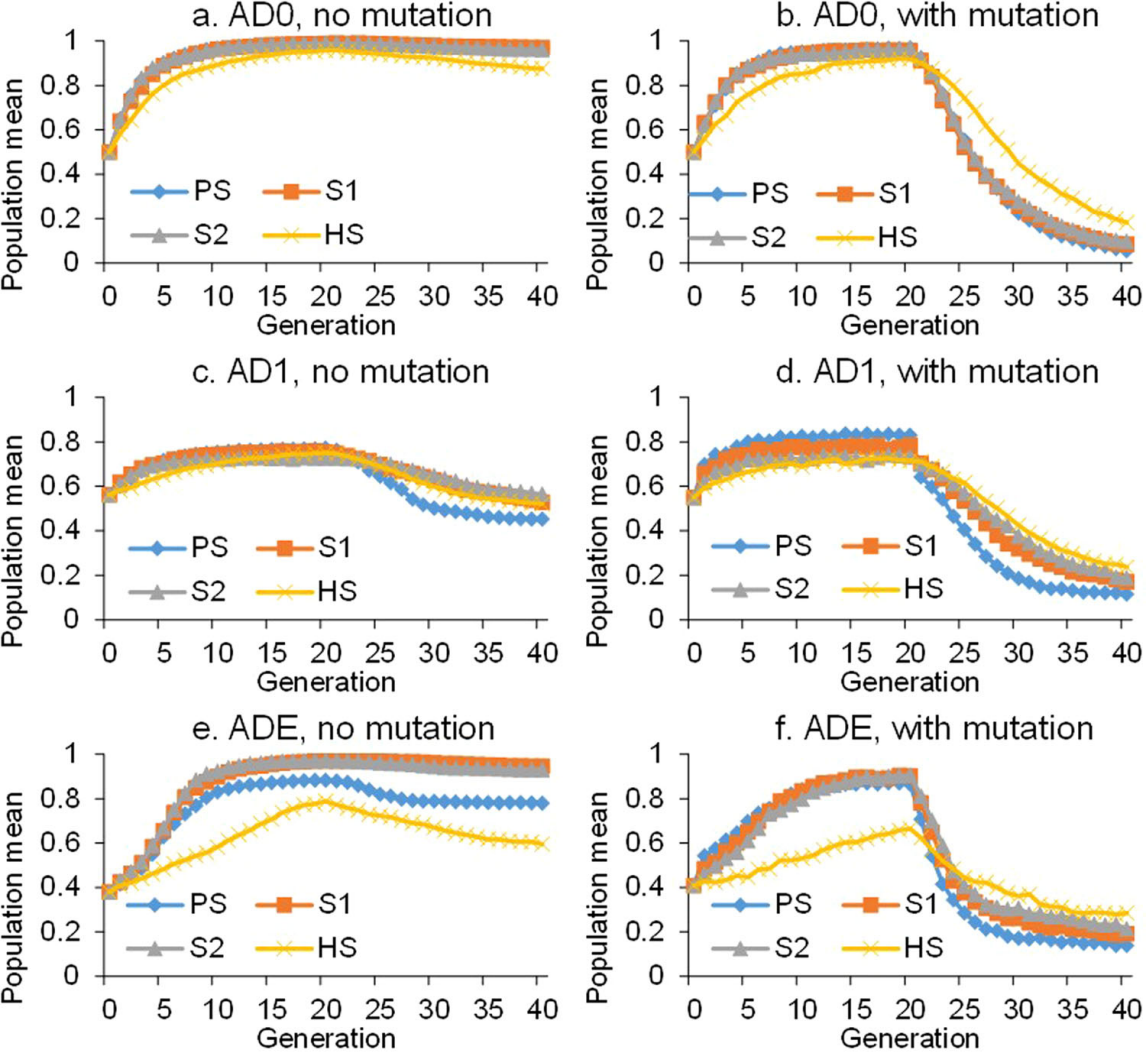
Population mean during 20 cycles of forward selection and 20 cycles of reverse selection. Three biallelic genetic models were considered, i.e., AD0 (additive model), AD1 (additive and dominant model) and ADE (additive, dominant and epistatic model). Four selection methods are simulated, i.e., phenotypic selection (PS), S1 family selection (S1), S2 family selection (S2), and half-sib selection (HS). **a** Model AD0 and no mutation was considered. **b** Model AD0 and mutation was considered. **c** Model AD1 and no mutation was considered. **d** Model AD1 and mutation was considered. **e** Model ADE and no mutation was considered. **f** Model ADE and mutation was considered.

The simulation was repeated for 100 runs. Figure 2 shows the change in population mean during 40 cycles of recurrent selection for the three effect models under no mutation and with mutation. The values shown in Fig. 2 have been adjusted by the lowest and highest genotypic values in each genetic model, and therefore range from 0 to 1. Classical quantitative genetics theory indicates that genetic gain from selection depends only on additive variance in the population^23^, where higher additive variance results in greater response to selection. Therefore, the population quickly moves to the peak trait value after a few cycles of selection under model AD0 with only the additive effects (Fig. 2a, b). At the peak trait value, the population is fixed for one homozygous genotype, leaving no genetic variation, and therefore, the reverse selection cannot act without mutations (Fig. 2a). This phenomenon has been well explained by the pure-line theory in breeding. However, when mutation occurs, the population quickly moves in the opposite direction as reverse selection is applied (Fig. 2b). Nonadditive variances are included in models AD1 and ADE, which reduce the efficiency of selection (Fig. 2c–f). Obviously, the population can never reach the highest genotypic value when nonadditive effects are present, and the individual with the highest performance is heterozygous. One peak trait value can still be reached, but diversity still occurs in the populations at the peak trait value. Therefore, when reverse selection is applied, populations can move in the opposite direction (Fig. 2c, e). This clearly indicates that mutation can make the selection more efficient under both models AD1 and ADE (Fig. 2d, f), which may be worth investigating further.

In Fig. 2, efficiency can be compared for the four selection methods as well. In model AD0, PS, S1 and S2 are almost equally efficient, but HS is less efficient in changing the population mean. This can be explained by the quantitative genetic theory that 100% of the additive variance is included in PS, S1, and S2, but 25% of the additive variance is included in HS^23^. For model AD1 without mutation, the difference among the four selection methods is much smaller during the 20 cycles of forward selection. However, PS is more efficient during the 20 cycles of reverse selection (Fig. 2c). For model AD1 with mutation, PS is more efficient than the other three methods during the 40 cycles of forward and reverse selections (Fig. 2d). For model ADE, S1 and S2 are more efficient during the 20 cycles of forward selection; HS is less efficient regardless of the presence of mutation (Fig. 2e, f).

Theories in classical quantitative genetics are mainly built on the multifactorial hypothesis. Some observations in Fig. 2 can be properly explained by the equation of genetic gain in quantitative genetics^23^. There are also some interesting issues that may be worthy of further investigation, such as the efficiency of selection when multiple alleles are considered in genetic models. In addition to the change in population mean as shown in Fig. 2, allelic frequencies, components of genetic variance, narrow- and broad-sense heritability, correlations between phenotypic traits, and correlations between environments can be investigated as well during short or long term selection, which cannot easily be studied theoretically. In addition, Blib is able to simulate a large number of genetic models and the outcomes of these models after long-term selection. By comparing the simulation results with observations from artificial experiments, such as long-term selection on oil and protein contents in corn^31^, the genetic architecture of quantitative traits may be better understood.

### Case study IV: Choice of testers in hybrid breeding programs

As the major component and activity in hybrid breeding programs, a large number of newly developed inbred lines are evaluated by their testing crosses with a few testers from the alternative heterotic group. The elite inbred lines thus selected are expected to have high combining ability, and are then used to develop new hybrids. However, the choice of suitable testers is an open question in hybrid breeding due to the complicated nature of the breeding procedure^32^. One Blib application module, called ISB-B4H, was developed to simulate breeding programs for developing hybrids as the final product in plants. A detailed description of ISB-B4H is beyond the scope of this paper. Instead, we use this module to show how the breeding efficiency yielded by different testers can be investigated by simulation in a more holistic view, by which the most suitable tester may be predicted and chosen for a particular hybrid breeding program.

Assume one hybrid breeding program, such as that for maize, begins with two heterotic groups, each consisting of 50 inbred lines as parents. One target trait, such as yield, is controlled by 100 biallelic loci distributed on 10 chromosomes with known genetic effects, which may come from genotype-to-phenotype modeling of historical breeding data, training populations or any other genetic studies^4,6,14^. In each breeding cycle, a total of 100 biparental crosses are made within one heterotic group, and from each cross a total of 100 doubled haploids (DH) are developed. Elite DH lines are selected through two stages of testcrossing evaluation. In stage one, the 10,000 DH lines are crossed with one tester from the other heterotic group, and 5% are selected on the basis of their performance in testing crosses. In stage two, the 500 DH lines retained from stage one are crossed again with the same tester, and 10% are selected, resulting in 50 lines which are then used to develop new hybrids and perform the next round of biparental crosses. The simulation was repeated for 100 runs.

**Fig. 3:**
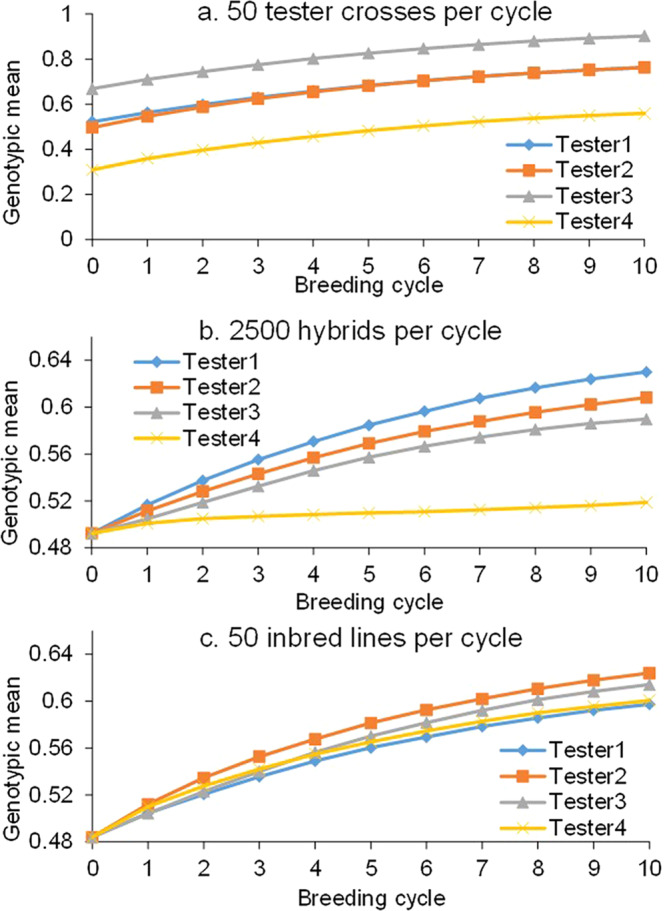
Genotypic means of tester crosses, hybrids with one other heterotic group, and inbred lines per se during 10 cycles of hybrid breeding. **a** Genotypic means of 50 tester crosses. **b** Genotypic means of 2500 hybrids with one other heterotic group. **c** Genotypic means of 50 inbred lines per se. The ordinate axes of the three subfigures are different for a better illustration of the differences among the four testers.

During simulation, the 50 retained DH lines at the end of each breeding cycle are evaluated by the 50 crosses with the tester, 2500 single crosses with the 50 inbred lines in the other heterotic group, and the inbred lines per se. Figure 3 shows the change in genotypic means from four testers in 10 breeding cycles. Tester1 and Tester2 are randomly selected from the 50 inbred lines in the other heterotic group; Tester3 harbors all favorable alleles at the 100 genetic loci; Tester4 harbors all unfavorable alleles at the 100 genetic loci. In this breeding program, selection is conducted solely on testing cross performance. The genotypic mean of testing crosses is advanced in parallel for the four testers (Fig. 3a). Tester3 has the largest number of favorable alleles, and the genotypic mean of its testing crosses is also the highest; Tester4 has the fewest favorable alleles, and the genotypic mean of its testing crosses is also the lowest. Due to the equal intensity of selection, genetic gains in testing crosses are equal among the four testers.

In hybrid breeding, the intensity of selection mostly comes from the selection on testing cross performance. However, the improvement of testing cross performance is in fact not the most important objective. Only a higher genotypic mean of single crosses with the alternative heterotic group can give breeders a greater opportunity to select more elite hybrids. Regarding the genotypic mean of 2500 single crosses, Tester1 is identified to be the best, and Tester4 is the worst (Fig. 3b). Genetic advances in crosses with the other heterotic group cannot easily be evaluated in practice but can be properly performed by the Blib application module ISB-B4H. In addition, genetic advances in inbred lines per se can also be determined (Fig. 3c). Advances observed in Fig. 3b, c are also called the in-direct or correlated genetic gains according to quantitative genetics theory^23^. In this sense, hybrid breeding actually provides an example where the in-direct gain outperforms the direct gain.

## Discussion

Predictions are expected at almost every stage during the rather long breeding procedure. When making crosses, breeders wish to know which parents should be chosen, and how many crosses should be made. In early segregating generations, breeders wish to know how many individuals should be planted in the field, how strong selection should be, and which phenotypic traits should be used as the selection criteria. At the stage of yield and adaptation testing, breeders wish to know the best locations for conducting the trials, how many locations should be used and how many replications should be arranged. Some issues of interest in breeding program design and analysis are beyond the reach of empirical experimental investigations. Simulation studies can be conducted to complement, extend, and improve the design and interpretation of empirical studies^2,7,10,33^.

Generally, decisions can be made from predictions; predictions can be made from simulations. With the accumulation of our knowledge and understanding of the inheritance of breeding traits, it is anticipated that the simulation, prediction and decision-supported tools will be in high demand^3,33^. Once properly designed and developed, these tools can help investigate many what-if crossing and selection scenarios, allow many scenarios to be tested and compared in silico in a short period of time, and finally help breeders make important decisions before conducting resource-demanding field experiments^7,11^. Simulation and prediction tools are essential components especially for the incoming breeding 4.0^34^, when breeding activities will be more driven by information technology in acquiring phenotypic data, molecular technology in acquiring genotypic data, and artificial intelligence in utilizing big data to make selection decisions.

We designed and developed Blib as reported in this study over our decades of research experience and advances in genetic analysis methodology^35–40^, breeding modeling and simulation^7,10–12^, and computer tool and software development^41–44^. Blib is composed of one global variable to define the generalized genetic models, one derived data type to define the genetics/breeding populations, and a number of general subroutines for manipulation and calculation. Blib has been well-developed and widely tested in recent years, and it can be treated as a computing and programming library for the purpose of genetics and breeding simulation. By using the library as the underlying components, various application modules can be readily developed to complete specific tasks, which have been shown by the four case studies. The separation of application modules from the library can save a great deal of time and effort when other researchers wish to develop new simulation tools or modify the existing modules.

In addition to genetic effects and models that have been considered in QU-GENE^5,6^, Blib can define and handle mutations (both for cytoplasm and nuclear genes), cytoplasm effects, cytoplasm by nuclear gene interactions, and fertilities of female and male gametes. By considering the additional genetic effects and models not included in QU-GENE, we expect that the application modules built on Blib will have greater potential for the theoretical genetic studies and breeding practice. For example, theories on population genetics seldom consider more than two factors simultaneously due to the intractability in mathematical deduction. By using the Blib platform, an application module can be easily developed to evaluate the joint effect of multiple factors affecting population structure, such as drift, mutation, migration, selection, stratification, and admixture. Such a module can also be used to generate a large number of genetic populations to evaluate the efficiency of statistical methods in genetic linkage mapping^40,41,44^ and genome-wide association studies (GWASs), and evaluate the efficiency of various prediction models in GS^4,14,33^.

We do not expect one or just a few application modules to be able to meet most purposes. In contrast, we expect that different application modules will need to be designed and developed for different purposes. However, one module can work with different models and simulate different genetic phenomena as defined in external input files. For demonstration and validation, we developed one Blib application module to simulate genetic drift in randomly mating populations (i.e., Case studies I and II), one module to simulate mass and family recurrent selection (i.e., Case study III) and one module to simulate breeding programs for developing hybrids (i.e., Case study IV). As shown in Case studies I and II, by modifying the input information, the module DRIFT can simulate not only the randomly mating populations under genetic drift but also the populations under the joint action of mutation and drift. It can also simulate a single locus with two alleles, a single locus with multiple alleles, and multiple loci with two or multiple alleles. When multiple loci are considered in the genetic model, linkage can be defined as well. A similar situation is true for the PRS module shown in Case study III, when different kinds of genetic effects are defined in input files. By adopting this philosophy, we believe that the need to develop new modules can be minimized. For most researchers, their efforts can be spent more effectively in building suitable genetic models, designing simulation experiments, running suitable application modules, and analyzing simulation results.

As another example, one specific module was developed to simulate the genetic recombination events between parents with the designed crossing schemes, which has been integrated with the genomic prediction tool to conduct genomic cross prediction in flax linseed breeding^45^. More application modules are being developed or planned, such as the in silico simulation of general breeding programs for developing pure lines, hybrids (as shown in Case study IV), and clonal varieties in plants, cross performance prediction and parental selection given the training populations with genotypic and phenotypic values, and simulation of various mapping populations from the user-defined linkage maps and genetic models. These modules are applicable to genetic studies and breeding practices in both sexually and asexually propagated species. Once developed and validated, all Blib-based application modules will become freely available to scientific researches.

To run one application module of Blib, at least three kinds of external files are needed as the input information. The first contains the information used to define the generalized genetic model (i.e., Gmodel), the second defines one or several initial populations, and the third defines the parameters in the simulation experiment. Although many genetic effects and models can be handled in Blib, not all of them have to be included in each external file or each simulation experiment. Take the application module used to simulate recurrent selections (i.e. Case study III) as an example. Three genetic models (i.e., pure additive, both additive and dominant, and epistasis) are defined separately in three external files. To run the same module on the three genetic models sequentially, we can monitor the change in population mean due to phenotypic and family selection schemes, as shown in Case study III. By adopting this philosophy, we believe that the procedure used to develop the Blib application module can be greatly simplified and more straightforward. The development of application modules can only focus on the third kind of input information and organization of the Blib subroutines to complete more specific and complex tasks.

To facilitate access to Blib and its application modules, we are currently developing some user interfaces that will help prepare the external files of input information for the global variable Gmodel and initial populations, which are fundamental to the simulation studies based on Blib. Alternatively, the information needed for simulation may be extracted from the existing database systems that are affiliated with on-going breeding programs^46^. By connecting with the information systems and ongoing breeding pipelines, the simulation platform Blib and its application modules can be used to conduct real-time simulation and prediction related to the outcomes of a large number of potential selection and crossing schemes. Thus, the optimum selection and crossing schemes can be identified and recommended to breeders before conducting field experiments. By doing so, simulation tools and approaches that are built on Blib are expected to make significant contributions by facilitating the change in conventional breeding from artificial selection to intelligent selection or helping move from the current stage to the incoming Breeding 4.0.

## Methods

### The global variable Gmodel for the generalized genetic models

**Fig. 4:**
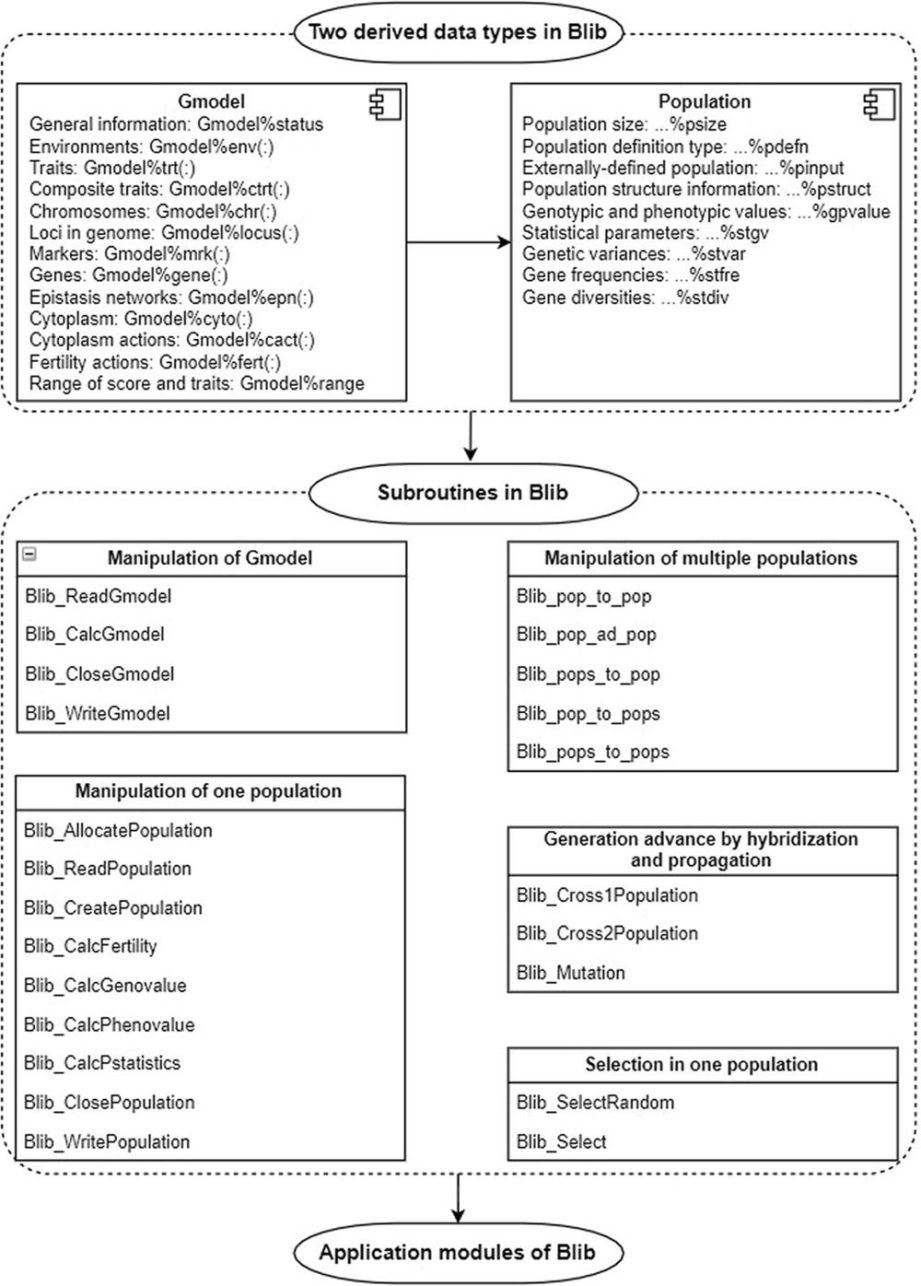
Relationships between derived data types and subroutines in Blib. Shown in the upper left is the definition of global variable Gmodel, which holds the required information on genetic models, e.g. environments, traits, composite traits, chromosomes, markers, genes, epistasis networks, cytoplasm, cytoplasm actions, and fertility actions. These information is packed up into 13 derived data types in Fortran. Shown in the upper right is one derived data type in Fortran, which can be called to define the genetic and breeding populations whenever needed. It consists of two integer variables, and seven variables of other derived data types. Based on Gmodel and the derived type of populations, subroutines are then developed, which are indicated in the lower part of the figure. Four subroutines are designed to manipulate the global variable Gmodel, and nine are designed to manipulate individual populations. Five subroutines are developed to implement the overloading features of populations, three are developed for the purpose of generation advancement, and two are developed for the purpose of selection.

In Blib, a global variable named Gmodel was first defined, which holds all necessary information in a generalized genetic model. This information was packed up into 13 derived data types in Fortran (Fig. 4; Supplementary Table 1). General information in Gmodel includes the numbers of environments, traits, composite traits, chromosomes, genetic loci (can be either genes or markers) in the genome, markers, genes, epistasis networks, cytoplasm, cytoplasm actions, and fertility actions. Included in the environmental information are the environmental name and frequency of occurrence in the target population of environments (TPE). Included in the trait information are the trait name, heritability and error variance in each environment. In addition, genes, epistasis networks, and cytoplasm actions for each trait are also stored to assist in the calculation of genotypic values for traits during simulation. Composite traits are calculated by addition, subtraction, multiplication and division from two or more underlying traits defined in Gmodel%trt(:) (Supplementary Table 1). The inclusion of composite traits in Blib also provides the opportunity to acquire the phenotypic values externally, such as the prediction of breeding values by one genomic prediction model that has been previously established in a training population.

Given next is the information on chromosomes and genetic loci on chromosomes (Supplementary Table 1). For each chromosome, the variable ‘Gmodel%chr(:)’ defines the chromosome name, and number of loci on the chromosome. For each set of ordered genetic loci on chromosomes, the variable ‘Gmodel%locus(:)’ defines the locus name, chromosomal position in centi-Morgans (cM), recombination frequency with the prededing locus, number of alleles at the locus, whether the locus is a marker or a gene, whether to consider mutation at the locus, and mutation rates if mutation is considered. For each locus specified as a marker, variable ‘Gmodel%mrk(:)’ defines the locus identity, and scores of all genotypes at the marker locus, allowing selection based on marker scores. For each locus specified as a gene, ‘Gmodel%gene(:)’ defines the locus identity, the number of traits affected, which traits are affected, and the values of all genotypes at the gene locus for each affected trait in each environment. For each epistasis network, the variable ‘Gmodel%epn(:)’ defines the trait identity, the environment identity, the number of loci in the network, which loci are involved, and the values of all genotypic combinations in the network.

Included in the cytoplasm information are the cytoplasm name, whether to consider mutation, and mutation rates if mutation is considered (Supplementary Table 1). Cytoplasm may have independent effects on some traits, or may interact with some nuclear genes to affect one trait. The effects on traits involving the cytoplasm are called cytoplasm actions in Blib. For each cytoplasm action, the variable ‘Gmodel%cact(:)’ defines the cytoplasm identity, the trait identification, the environment identity, the number of loci in the action, which nuclear genetic loci are involved, and the values of all genetic combinations in the action (Supplementary Table 1). The fertility of female and male gametes may be affected by the cytoplasm alone, nuclear genes alone, or cytoplasm by nuclear gene interactions. These effects on female and male fertility are called fertility actions in Blib. For each fertility action, the variable ‘Gmodel%fert(:)’ defines the cytoplasm identification, the environment identity, the number of loci in the action, which loci are involved, and the values of all genotypic combinations in the action (Supplementary Table 1). Finally, the variable ‘Gmodel%range’ defines the lowest and highest marker scores and the lowest and highest values for each trait (Supplementary Table 1), by which the phenotypic traits may be adjusted to be located in the range of 0 to 1 for the convenience of comparison.

### Technical details of key variables included in Gmodel

Suitable definition of the large number of highly varied and complicated genetic models is key in developing the genetic simulation tools or platforms. More technical details of the five selected variables are given below in Supplementary Table 1, which consist of the core contents of the global variable Gmodel. When loading the information from one external file, the pointer ‘Gmodel%locus(:)’ is first allocated, with the size being equal to the number of genetic loci defined in Gmodel%status. For each ordered genetic locus defined by TYPE(Gmodel_locus) (Supplementary Fig. 1), one character variable, i.e., ‘Gmodel%locus(:)%name’, represents name of the locus. Two real variables, i.e., ‘Gmodel%locus(:)%pos’ and ‘Gmodel%locus(:)%recfreq’, represent the chromosomal position in cM and the recombination frequency with the preceding locus. Three integer variables, i.e., ‘Gmodel%locus(:)%nallel’, ‘Gmodel%locus(:)%isgene’ and ‘Gmodel%locus(:)%mutate’, represent the number of alleles at the locus, whether the locus is a marker or a gene, and whether or not to consider mutation at the locus. The last real variable, i.e., ‘Gmodel%locus(:)%murate’, is defined as a real pointer of one-dimensional array, which is to be allocated and assigned with mutation rates when mutation is considered. Input information of seven loci is given in the lower part of Supplementary Fig. 1 as an illustration. For example for the second locus, we have Gmodel%locus(2)%name = ‘Locus2’ and Gmodel%locus(2)%pos = 10.0, and therefore, its genetic distance from the preceding locus is equal to 10 cM from which variable Gmodel%locus(2)%recfreq for recombination frequency can be assigned by Haldane’s mapping function. Gmodel%locus(2)%nallel = 3 indicates that there are three alleles at the locus; Gmodel%locus(2)%isgene = 0 indicates that the locus is a marker; and Gmodel%locus(2)%mutate = 1 indicates that mutation is considered at the locus. Then, Gmodel%locus(2)%murate is defined as an array with a size of 6, and assigned the six mutation rates from allele 1 to alleles 2 and 3 (i.e., 0.0001 and 0.0002), from allele 2 to alleles 1 and 3 (i.e., 0.0003 and 0.0004), and from allele 3 to alleles 1 and 2 (i.e., 0.0005 and 0.006) (Supplementary Fig. 1).

The pointer ‘Gmodel%gene(:)’ is first assigned with the size being equal to the number of genes defined in Gmodel%status. For each gene defined by ‘TYPE(Gmodel_gene)’ (Supplementary Fig. 2), two integer variables, i.e., ‘Gmodel%gene(:)%wlocus’ and ‘Gmodel%gene(:)%ntrt’, represent the locus identity and number of traits affected by the gene. The integer variable ‘Gmodel%locus(:)%wtrt’ is defined as a pointer, which is to be allocated and assigned with the trait identity. The real variable ‘Gmodel%locus(:)%gvalue(:,:,:)’ is defined as a pointer of three dimensions, which is to be allocated and assigned with the genotypic values of all genotypes at the gene locus for all affected traits in all environments. For example for the gene defined in the lower part of Supplementary Fig. 2, Gmodel%gene(1)%wlocus = 3, and Gmodel%gene(1)%ntrt = 2, indicating that the gene is located at the third locus in the genome and has effects on two traits. The real variable ‘Gmodel%locus(1)%gvalue’ is defined as an array of three dimensions with sizes of 2, 3, and 10, representing the number of traits affected by the gene, number of environments, and number of genotypic combinations at the gene locus. The 60 values shown on the lower right side in Supplementary Fig. 2 represent the 10 genotypic values for the second and third traits in the three environments which are defined by Gmodel%env(:).

The pointer ‘Gmodel%cyto(:)’ is first defined, with the size being equal to the number of cytoplasms defined in Gmodel%status. For each cytoplasm defined by ‘TYPE(Gmodel_cyto)’ (Supplementary Fig. 3), one character variable, i.e., ‘Gmodel%cyto(:)%name’, represents the name of the cytoplasm. One integer variable, i.e., ‘Gmodel%cyto(:)%mutate’, represents whether or not to consider mutation for the cytoplasm. One real variable, i.e., ‘Gmodel%locus(:)%murate’, is defined as a pointer that is to be allocated and assigned with mutation rates if mutation is considered. For example, for the fourth cytoplasm in the lower part of Supplementary Fig. 3, Gmodel%cyto(4)%name = ‘Cyto4’, and Gmodel%cyto(4)%mutate = 1, indicating that mutation is considered for the cytoplasm. Gmodel%cyto(4)%murate is defined as an array with a size of 4, and assigned the four mutation rates from cytoplasm 4 to cytoplasms 1, 2, 3 and 5 (i.e., 0.0090, 0.0050, 0.0000, and 0.0000) (Supplementary Fig. 3).

The pointer ‘Gmodel%cact(:)’ is first defined with the size being equal to the number of cytoplasm actions defined in Gmodel%status. For each cytoplasm action defined by ‘TYPE(Gmodel_cact)’ (Supplementary Fig. 4), five integer variables, i.e., ‘Gmodel%cact(:)%wcyto’, ‘Gmodel%cact(:)%wtrt’, ‘Gmodel%cact(:)%wenv’, ‘Gmodel%cact(:)%nvalue’, and ‘Gmodel%cact(:)%nlocus’ represent the cytoplasm identity, trait identity, environment identity, number of values, and number of loci included in the action, respectively. The number of values, i.e., ‘Gmodel%cact(:)%nvalue’, is automatically calculated from the number of alleles at each locus included in the action. The integer variable ‘Gmodel%cact(:)%wlocus’ is defined as an integer pointer, which is to be allocated and assigned the locus identity. The real variable ‘Gmodel%cact(:)%cvalue(:)’ is defined as a real array pointer, which is to be allocated and assigned the genotypic values in the action. For example for the first action defined in the lower part of Supplementary Fig. 4, Gmodel%cact(1)%wcyto = 2, Gmodel%cact(1)%wtrt = 4, Gmodel%cact(1)%wenv = 1, Gmodel%cact(1)%nvalue = 1, and Gmodel%cact(1)%nlocus = 0, indicating that the second cytoplasm has an independent effect on the fourth trait in the first environment. The independent effect is equal to 1.01, which is assigned to the allocated real array ‘Gmodel%cact(2)%cvalue(1:1)’. For the second action in Supplementary Fig. 4, the first cytoplasm and the tenth locus together affect the first trait in the first environment. There are two alleles at the tenth locus (Supplementary Fig. 1); therefore, three genotypic values are given in Supplementary Fig. 4 for the action, which are assigned to the allocated real array ‘Gmodel%cact(2)%cvalue(1:3)’. For the third action in Supplementary Fig. 4, the third cytoplasm and three loci together affect the fourth trait in the first environment. The three loci (i.e., 10, 11, and 22) have 2, 2, and 3 alleles (Supplementary Fig. 1), resulting in 3, 3, and 6 genotypes, respectively. Therefore, a total of 54 genotypic values are given in Supplementary Fig. 4 for the action, which are assigned to the allocated real array ‘Gmodel%cact(3)%cvalue(1:54)’.

We assume that fertility is independent of environment. Alternatively, fertility is mainly controlled by cytoplasm and/or nuclear genes under normal environmental conditions. The pointer ‘Gmodel%fert(:)’ is first defined, with the size being equal to the number of fertility actions defined in Gmodel%status. For each fertility action defined by ‘TYPE(Gmodel_fert)’ (Supplementary Fig. 5), three integer variables, i.e., ‘Gmodel%fert(:)%wcyto’, ‘Gmodel%fert(:)%nvalue’, and ‘Gmodel%fert(:)%nlocus’, represent the cytoplasm identity, number of values, and number of loci included in the action, respectively. The number of values, i.e., ‘Gmodel%fert(:)%nvalue’, is automatically calculated from the number of alleles at each locus included in the action. The integer variable ‘Gmodel%fert(:)%wlocus’ is defined as a pointer, which is to be allocated and assigned with the locus identity. Two real variables ‘Gmodel%cact(:)%fvalue(:)’ and ‘Gmodel%cact(:)%mvalue(:)’, are defined as real pointers of a one-dimensional array, which are to be allocated and assigned with the female and male fertility values. For example for the first action defined in the lower part of Supplementary Fig. 5, Gmodel%fert(1)%wcyto = 3, Gmodel%fert(1)%nvalue = 1, and Gmodel%fert(1)%nlocus = 0, indicating that the third cytoplasm has an independent effect on fertility. The female fertility is equal to 1.00 (i.e., normal), which is assigned to the allocated array ‘Gmodel%fert(1)%fvalue(1:1)’; the male fertility is equal to 0.90 (i.e., partial sterility), which is assigned to the allocated array ‘Gmodel%fert(1)%mvalue(1:1)’. For the second action in Supplementary Fig. 5, Gmodel%fert(2)%wcyto = 0, Gmodel%fert(2)%nvalue = 6, and Gmodel%fert(2)%nlocus = 1, indicating that there is one locus with independent effects on fertility, i.e., locus 22. There are three alleles at the locus (Supplementary Fig. 1); therefore, six values are given for female fertility, which are assigned to the allocated array ‘Gmodel%cact(2)%fvalue(1:6)’; and six values are given for male fertility, which are assigned to the allocated array ‘Gmodel%cact(2)%mvalue(1:6)’. For the third action in Supplementary Fig. 5, Gmodel%fert(3)%wcyto = 0, Gmodel%fert(3)%nvalue = 18, and Gmodel%fert(3)%nlocus = 2, indicating that two loci together affect fertility, i.e., locus 1 and locus 2. There are two alleles at locus 1 and three alleles at locus 2 (Supplementary Fig. 1), resulting in a total of 18 (i.e., 3×6) genotypes when the two loci are considered together. Therefore, 18 values are given for female fertility, which are assigned to the allocated array ‘Gmodel%cact(3)%fvalue(1:18)’, and 18 values are given for male fertility which are assigned to the allocated array ‘Gmodel%cact(3)%mvalue(1:18)’.

### Definition of the generalized genetic and breeding populations in Blib

In Blib, ‘Population’ is one derived data type in Fortran, which can be called to define the generalized genetic and breeding populations whenever needed (Supplementary Fig. 6). For example, the code ‘TYPE(Population):: Popg, Popb, PopF1(3)’ defines two populations (i.e., Popg and Popb), and one array of populations with the size of three, i.e., PopF1(1:3), which can then be manipulated in Blib subroutines and application modules. Type ‘Population’ consists of two integer variables, and seven variables of other derived data types (Fig. 1; Supplementary Table 2). One integer variable, i.e., ‘…%psize’, saves the population size, and the other one, i.e., ‘…%pdefn’, saves the method to generate the individual genotypes, e.g., by allele frequencies or by allele combinations. The second integer variable is mainly used when assigning one population from one user-defined external input file (Supplementary Table 2).

The variable ‘…%pinput’ saves the frequencies of cytoplasm, and frequencies of alleles at each locus, or the specified allele combinations, which are loaded externally (Supplementary Table 2). The variable ‘…%pstruct’ saves the parental identity, cytoplasm type, and two alleles at two homologous chromosomes of every diploid individual in the population. This is the most important piece of information regarding the genetic and breeding populations. The variable ‘…%gpvalue’ holds the female and male gametic fertilities, marker score, and genotypic and phenotypic values of every diploid individual in the population, which are allocated and assigned whenever needed in application modules.

The other variables of derived data types record the genetic parameters at the population level, which are allocated and assigned upon request. The variable ‘…%stgv’ records population means, variances, genotype by environment interactions, broad-sense heritability, trait correlations, and environmental correlations. The variable ‘…%stvar’ records the additive and dominance variances, and broad-sense and narrow-sense heritabilities. The variable ‘…%stfre’ records the frequencies of cytoplasm, and frequencies of alleles at each marker or gene locus. The variable ‘…%stdiv’ records the diversities of markers and genes. Frequencies and diversities can also be calculated independently from genes located on each chromosome, from genes affecting each trait, or from genes in each epistasis network. All these parameters have been widely used in theoretical and applied population genetics.

### Major Fortran subroutines in Blib

Based on the previously described global variable Gmodel and the derived data type ‘Population’, a number of general subroutines were then developed for specific purposes (Fig. 1; Supplementary Table 3). Four subroutines were designed to manipulate the global variable Gmodel (1.1 to 1.4 in Supplementary Table 3), such as read the user-defined information to Gmodel from an external file (i.e., Blib_ReadGmodel), calculating and assigning the variables in Gmodel not directly given in external file (i.e., Blib_CalcGmodel), releasing the allocated memory in Gmodel (i.e., Blib_CloseGmodel), and writing the information in Gmodel to an external file (i.e., Blib_WriteGmodel).

Nine subroutines were designed to manipulate the population (Fig. 1; 2.1 to 2.9 in Supplementary Table 3). The first three were designed to allocate memory to the to-be-used variables in the population (i.e., Blib_AllocatePopulation), read one population from one user-defined external file (i.e., Blib_ReadPopulation), and create the genotypes of all individuals from the user-defined cytoplasm frequency and allele frequencies (i.e., Blib_CreatePopulation). The three subroutines in the middle were designed to calculate female and male fertility values for each individual in the population (i.e., Blib_CalcFertility), calculate genotypic values for each individual in the population for a specific trait in a specific environment (i.e., Blib_CalcGenovalue), and calculate phenotypic values for each individual in the population for a specific trait in a specific environment (i.e., Blib_CalcPhenovalue). Subroutine Blib_CalcPstatistics calculates the statistical parameters related to the genetic and breeding population, such as population means for marker score and traits, additive and dominance variances, frequencies of alleles at each locus, and genetic diversities. The last two can be used to release part or all of the allocated memory in the population (i.e., Blib_ClosePopulation), and write one population to an external file (i.e., Blib_WritePopulation).

Overloading is an important feature in modern programming languages. Five subroutines were designed to have the overloading features for populations (3.1–3.5 in Supplementary Table 3). By using these subroutines, we can define one population as an empty population (i.e., Blib_pop_to_pop), merge two populations to make a new population (i.e., Blib_pop_ad_pop), merge a one-dimensional array of populations to obtain a new population (i.e., Blib_pops_to_pop), split one population into a one-dimensional array of populations each with a size of one (i.e., Blib_pop_to_pops), and assign a one-dimensional array of populations to another one-dimensional array of populations (i.e., Blib_pops_to_pops). By calling the overloading subroutines, the development of Blib application modules can be greatly simplified, especially when considering that both among-family selection and within-family selection are frequently applied in most breeding programs.

Three subroutines were developed for the purpose of generation advancement (4.1 to 4.3 in Supplementary Table 3). The first one, i.e., Blib_Cross1Population, was designed to conduct hybridization using one population of parents, and then generate a new population of progenies. The second one, i.e., Blib_Cross2Population, was designed to conduct the hybridization between two populations (one used as female parents, and the other used as male parents), and then generate a new population of progenies. The third one, i.e., Blib_Mutation, was designed to conduct mutation (both cytoplasm and the alleles at each mutating locus) once a new population is formed. Notably, the third subroutine is always automatically called by the first two.

Two subroutines were developed for the purpose of selection (5.1 to 5.2 in Supplementary Table 3). One was designed to randomly select a given number of individuals from one population, and form a new population (i.e., Blib_SelectRandom). The other one was designed to select a given number of individuals by one specified selection mode from one population (i.e., Blib_Select), such as top selection (i.e., individuals with the highest trait values are selected), bottom selection (i.e., individuals with the lowest trait values are selected), and middle selection (i.e., individuals with medium trait values are selected). More advanced selection methods are sure to occur, which will be considered as Blib application modules are further developed.

### Statistics and reproducibility

All statistical analyses were performed using Microsoft Excel 2016. Case studies were conducted using the application modules and input files provided in Supplementary Software 1. In case studies I and II, two population sizes were set at 10 and 50. In case study III, size of the initial population was set at 100. In case study IV, each of the two heterotic groups consisted of 50 inbred lines. Each case study was repeated for 100 simulation runs as defined in the input files.

### Reporting summary

Further information on research design is available in the Nature Research Reporting Summary linked to this article.

## Supplementary information

Supplementary Tables and Figures

Description of Additional Supplementary Files

Supplementary Data 1

Supplementary Software 1

Reporting Summary

## Data Availability

Input and output files of the four case studies were provided as Supplementary Software 1. Source data underlying figures were presented in Supplementary Data 1.

## Code availability

Compiled codes of application modules used in the four case studies were provided as Supplementary Software 1.
