# Supplementary material for: Blib is a multi-module simulation platform for genetics studies and intelligent breeding
Source: Commun Biol. 2022 Nov 3;5:1167. doi: 10.1038/s42003-022-04151-9 (PMC9630530; doi:10.1038/s42003-022-04151-9)
Supplement: Supplementary file 1 — Supplementary Tables and Figures [file 42003_2022_4151_MOESM1_ESM.pdf]

Supplementary Table 1 Thirteen derived data types included in the global variable Gmodel

No.	Information	Variable	Contents of the stored information
1	General	Gmodel%status	Numbers of environments, traits, composite traits, chromosomes, genetic loci, markers, genes, epistasis networks, cytoplasm, cytoplasm actions, and fertility actions et al.
2	Environments	Gmodel%env(:)	Defined for each environment are environment name and frequency of occurrence in the target population of environments (TPE). Number of environment is given by Gmodel%status.
3	Traits	Gmodel%trt(:)	Defined for each trait are trait name, heritabilities and error variances in all environments defined by Gmodel%env(:). In addition, genes, epistasis networks, and cytoplasm actions on each trait are also stored to assist the calculation of genotypic values during simulation. Number of traits is given by Gmodel%status.
4	Composite traits	Gmodel%ctrt(:)	Defined for each composite trait are the way to derive the composite trait, number of underlying traits and their weights in the composite trait. Number of composite traits is given by Gmodel%status.
5	Chromosomes	Gmodel%chr(:)	Defined for each chromosome are chromosome name, and number of loci on the chromosome. Number of chromosomes is given by Gmodel%status.
6	Loci in genome	Gmodel%locus(:)	Defined for each ordered locus are locus name, chromosomal position in centi-Morgan (cM), recombination frequency with previous locus, number of alleles at the locus, whether the locus is a marker or a gene, whether to consider mutation at the locus, and mutation rates if mutation is considered. Total number of loci on all chromosomes is given by Gmodel%chr(:).

7	Markers	Gmodel%mrk(:)	Defined for each marker locus are the locus identification, and scores of all genotypes at the marker locus. Number of markers is determined by Gmodel%locus, which is also assigned to Gmodel%status.
8	Genes	Gmodel%gene(:)	Defined for each gene locus are the locus identification, number of traits affected, which traits are affected, and values of all genotypes at the gene locus for all affected traits in all environments. Number of genes is determined by Gmodel%locus, which is also assigned to Gmodel%status.
9	Epistasis networks	Gmodel%epn(:)	Defined for each epistasis network are the trait identification, environment identification, number of loci in the network, which loci are involved, and values of all genotypes in the network. Number of epistasis networks is given by Gmodel%status.
10	Cytoplasm	Gmodel%cyto(:)	Defined for each cytoplasm are cytoplasm name, whether to consider mutation, and mutation rates of the cytoplasm to other cytoplasm if mutation is considered. Number of cytoplasm is given by Gmodel%status.
11	Cytoplasm actions	Gmodel%cact(:)	Defined for each cytoplasm action are the cytoplasm identification, trait identification, environment identification, number of loci in the action, which loci are involved, and values of all genotypic combinations in the action. Number of cytoplasm actions is given by Gmodel%status.
12	Fertility actions	Gmodel%fert(:)	Defined for each fertility action are the cytoplasm identification, number of loci in the action, which loci are involved, and female and male fertility values of all genetic combinations in the action. Number of cytoplasm actions is given by Gmodel%status.
13	Range of score and	Gmodel%range	Defined in this variable are lowest and highest marker scores, and lowest and highest values

traits	for each trait as defined by Gmodel%trt(:).

Supplementary Table 2 Variables included in the ‘Population’ derived data type

No.	Information	Variable	Contents of the stored information
1	Population size	…%psize	Number of diploid individuals in the population.
2	Population definition	…%pdefn	Definition of individual genotypes in the population, i.e. by allele frequencies or by allele combinations.
3	Externally-defined (or base) population	…%pinput	Frequencies of cytoplasm and frequencies of alleles at each locus, or the specified allele combinations loaded externally.
4	Population structural information	…%pstruct	Parental identification, cytoplasm type, and alleles at two homologous chromosomes of every diploid individual in the population. This is the most important part of information on the population!
5	Genotypic and phenotypic values	…%gpvalue	Fertility values, marker score, genotypic values, or phenotypic values of every diploid individual in the population.
6	Population parameters	…%stgv	Population parameters on means, variances, genotype by environment interactions, broad-sense heritability, trait correlations, and environmental correlations.
7	Genetic variances	…%stvar	Population parameters on additive and dominant variances, and broad-sense and narrow-sense heritabilities.
8	Gene frequencies	…%stfre	Population parameters on frequencies of cytoplasm, markers, and genes.
9	Gene diversities	…%stdiv	Population parameters on diversities of markers, and genes.

Supplementary Table 3 Major subroutines implemented in Blib

Subroutine	Description of the major function
1. Manipulation of the global variable Gmodel	
1.1 Blib_ReadGmodel	Read the user-defined information to global variable Gmodel from an external file
1.2 Blib_CalcGmodel	Calculate and assign the variables in Gmodel which are not given in the external file
1.3 Blib_CloseGmodel	Release the allocated memory in global variable Gmodel
1.4 Blib_WriteGmodel	Write global variable Gmodel to an external file
2. Manipulation of one population	
2.1 Blib_AllocatePopulation	Allocate memory to the to-be-used variables in population
2.2 Blib_ReadPopulation	Read one user-defined population from an external file
2.3 Blib_CreatePopulation	Create the genotypes of all individuals from the user-defined cytoplasm and allele frequencies (i.e. …%pstruct)
2.4 Blib_CalcFertility	Calculate the female and male fertility values for each individual in the population (i.e. ...%gpvalue%fertility)
2.5 Blib_CalcGenovalue	Calculate the genotypic values for each individual in the population for one given trait and environment (i.e. ...%gpvalue)
2.6 Blib_CalcPhenovalue	Calculate the phenotypic values for each individual in the population for one given trait and environment (i.e. ...%gpvalue)
2.7 Blib_CalcPstatistics	Calculate the statistical parameters of a genetic/breeding population such as population means of marker score and traits (i.e. ...%stgv), additive and dominant variances (i.e. ...%stgv), frequencies of alleles at each locus (i.e. %stfre), and genetic diversities (i.e. ..%stdiv)
2.8 Blib_ClosePopulation	Release part or all of the allocated memory in population
2.9 Blib_WritePopulation	Write one population to an external file
3. Manipulation or overloading of multiple populations	
3.1 Blib_pop_to_pop	Assign one population to a new population
3.2 Blib_pop_ad_pop	Add up two populations to have a new population
3.3 Blib_pops_to_pop	Add up an array of populations to have a new population
3.4 Blib_pop_to_pops	Split one population to an array of populations, each with the size of one
3.5 Blib_pops_to_pops	Assign an array of populations to a new array of populations

4. Generation advance by hybridization and propagation	
4.1 Blib_Cross1Population	Conduct hybridization using one population as parents to generate a new population as progenies
4.2 Blib_Cross2Population	Conduct hybridization between two populations (one as female parents, and the other as male parents) to generate a new population as progenies
4.3 Blib_Mutation	Conduct mutation (both cytoplasm and alleles at each locus) once a new population is formed
5. Selection in one population	
5.1 Blib_SelectRandom	Select a given number of individuals randomly from one population and form a new population
5.2 Blib_Select	Select a given number of individuals by the specified mode from one population, i.e. such as top, bottom, and middle

```
TYPE Gmodel_locus
    CHARACTER*15  :: name      = 'Locus'
    REAL          :: pos       =  0.0
    REAL          :: recfreq   =  0.0
    INTEGER*1     :: nallel    =  2
    INTEGER       :: isgene    =  0
    INTEGER       :: mutate    =  0
    REAL, POINTER :: murate(:) => NULL()
END TYPE Gmodel_locus
```

```
!locus   name     wchr   pos     nallel   isgene   mutate murates…
1        Locus1   1      0.00    2        0        0
2        Locus2   1      10.00   3        0        1         0.0001   0.0002   0.0003
                                                            0.0004   0.0005   0.0006
3        Locus3   1      20.00   4        1        0
5        Locus5   1      30.00   3        0        0
10       Loc10    2      20.00   2        1        0
11       Loc11    2      30.00   2        1        0
22       Loc22    3      60.00   3        1        0
```

Supplementary Fig. 1 The derived data type used in Fortran to define one genetic locus (upper) and part of the input information at seven genetic loci (lower)

```
TYPE Gmodel_gene
    INTEGER          :: wlocus      =  1
    INTEGER          :: ntrt        =  1
    INTEGER, POINTER :: wtrt(:)       => NULL()
    REAL, POINTER    :: gvalue(:,:,:) => NULL()
END TYPE Gmodel_gene
```

```
!gene   wlocus   nallel   ntrt
!                wtrt     wenv     gvalue…
1       3        4        2
                 2        1        1.1 2.2 3.3 4.4 5.5 6.6 7.7 8.8 9.9 10.1
                          2        1.2 2.1 3.4 4.3 5.6 6.5 7.8 8.7 0.1 10.9
                          3        3.4 2.1 6.7 3.4 5.5 9.6 2.1 4.7 9.9  0.0
                 3        1        0.1 0.2 0.3 0.4 0.5 0.6 0.7 0.8 0.9  1.0
                          2        0.1 0.1 0.1 0.2 0.2 0.2 0.3 0.3 0.3  0.4
                          3        0.5 0.5 0.5 0.5 0.5 0.6 0.7 0.8 0.9  0.0
```

Supplementary Fig. 2 The derived data type used in Fortran to define one gene (upper) and part of the input information for genes (lower)

```
TYPE Gmodel_cyto
    CHARACTER*15  :: name      = 'Cyto'
    INTEGER       :: mutate    =  0
    REAL, POINTER :: murate(:) => NULL()
END TYPE Gmodel_cyto
```

```
!cyto   name    mutate  murates…
1       Cyto1   0
2       Cyto2   1       0.0012 0.0034 0.0200 0.0080
3       Cyto3   0
4       Cyto4   1       0.0900 0.0050 0.0000 0.0000
5       Cyto5   0
```

Supplementary Fig. 3 The derived data type used in Fortran to define one cytoplasm (upper) and part of the input information for the cytoplasm (lower)

```
TYPE Gmodel_cact
    INTEGER*1       :: wcyto     = 1
    INTEGER         :: wtrt      = 1
    INTEGER         :: wenv      = 0
    INTEGER         :: nvalue    = 0
    INTEGER         :: nlocus    = 0
    INTEGER, POINTER :: wlocus(:) => NULL()
    REAL, POINTER    :: cvalue(:) => NULL()
END TYPE Gmodel_cact
```

```
!cact    wcyto    wtrt    wenv    nlocus    wlocus…
!                                           cvalue…
1        2        4       1       0
                                            1.01
2        1        1       1       1         10
                                            1.11 1.22 1.33
3        3        4       1       3         10   11   22
                                            2.01 2.02 2.03 2.04 2.05 2.06 2.07 2.08 2.09
                                            2.10 2.11 2.12 2.13 2.14 2.15 2.16 2.17 2.18
                                            2.19 2.20 2.21 2.22 2.23 2.24 2.25 2.26 2.27
                                            2.28 2.29 2.30 2.31 2.32 2.33 2.34 2.35 2.36
                                            2.37 2.38 2.39 2.40 2.41 2.42 2.43 2.44 2.45
                                            2.46 2.47 2.48 2.49 2.50 2.51 2.52 2.53 2.54
```

Supplementary Fig. 4 The derived data type used in Fortran to define one cytoplasm action (upper) and part of the input information for cytoplasm actions (lower)

```
TYPE Gmodel_fert
    INTEGER*1        :: wcyto     = 0
    INTEGER          :: nvalue    = 0
    INTEGER          :: nlocus    = 0
    INTEGER, POINTER :: wlocus(:) => NULL()
    REAL, POINTER    :: fvalue(:) => NULL()
    REAL, POINTER    :: mvalue(:) => NULL()
END TYPE Gmodel_fert
```

```
!fert   wcyto   nlocus   wlocus
!                                 fvalue (female gamete fertility)
!                                 mvalue (male gamete fertility)
1       3       0
                                  1.00
                                  0.90
2       0       1        22
                                  0.90 0.90 0.90 1.00 1.00 1.00
                                  1.00 1.00 1.00 1.00 1.00 1.00
3       0       2        1        2
                                  1.0 1.0 1.0 1.0 1.0 1.0 1.0 1.0 1.0 1.0
                                  1.0 1.0 1.0 1.0 1.0 1.0 1.0 1.0
                                  0.1 0.1 0.1 0.2 0.2 0.2 0.0 0.0 0.0 0.3
                                  0.3 0.3 0.5 0.5 0.5 0.5 0.5 0.5
```

Supplementary Fig. 5 The derived data type used in Fortran to define one fertility action (upper) and part of the input information for fertility actions (lower)

```fortran
TYPE Population
    INTEGER :: psize = 0
    INTEGER :: pdefn = 3

    TYPE(Pop_popbase)   :: popbase
    TYPE(Pop_structure) :: pstruct
    TYPE(Pop_gpvalue)   :: gpvalue

    TYPE(Pst_genovalue) :: stgv
    TYPE(Pst_variance)  :: stvar
    TYPE(Pst_frequency) :: stfre
    TYPE(Pst_diversity) :: stdiv
END TYPE Population
```

Supplementary Fig. 6 The derived data type used in Fortran to define a generalized genetic and breeding population
