# Supplementary material for: Blib is a multi-module simulation platform for genetics studies and intelligent breeding
Source: Commun Biol. 2022 Nov 3;5:1167. doi: 10.1038/s42003-022-04151-9 (PMC9630530; doi:10.1038/s42003-022-04151-9)
Supplement: Supplementary file 2 — Description of Additional Supplementary Files [file 42003_2022_4151_MOESM2_ESM.docx]

1 **Description of Additional Supplementary Files**

2

3 **File name:** Supplementary Software 1

4 **Description:** The input files, output files, and application modules for the four case studies.

5 **File name:** Supplementary Data 1

6 **Description:** The source data used to generate the main figures.

7
