# Supplementary material for: Blib is a multi-module simulation platform for genetics studies and intelligent breeding
Source: Commun Biol. 2022 Nov 3;5:1167. doi: 10.1038/s42003-022-04151-9 (PMC9630530; doi:10.1038/s42003-022-04151-9)
Supplement: Supplementary file 4 — Supplementary Software 1 [file 42003_2022_4151_MOESM4_ESM.zip › Supplementary software/ReadMe.docx]

**Notes on running the Blib application modules in case studies**

1. **System requirements**

Three Blib application modules are used in the four case studies in the manuscript. Module “Drift.exe” was used in Case studies I and II, “PRS.exe” was used in Case study III, and module “ISB.exe” was used in Case study IV. These modules are 64 bit, running on MS Windows XP/Vista/7/10. For some computers, one additional MS Windows DLL called “libiomp5md.dll” may still be needed to run these modules.

1. **Installation guide**

These modules are command-line applications. There is no need for installation.

1. **Output messages after the running of each module**

Examples have been provided with the applications. Double click the exe files to run the applications.

In Case studies I and II, module Drift.exe simulates the genetic drift in random-mating populations. Four output files are generated after running. Output with extension name *.fre contains the allele frequency at each locus. Output with extension name *.fst contains Fst values at each locus. Output with extension name *.sum contains the allele numbers before and after random drift, as well as the numbers of individuals containing different alleles. Output with extension name *.ld contains LD values between two different loci.

In Case study III, module PRS.exe simulates various phenotypic recurrent selections. Three output files are generated after running. Output with extension name *.div contains the gene diversity at each locus. Output with extension name *.fre contains the allele frequencies at each locus. Output with extension name *.tgv contains some basic statistics of the population, such as population mean, genetic variance, adjusted population mean, heritability in broad sense, heritability in narrow sense, additive variance, and dominance variance.

ISB.exe simulates the breeding procedure for selecting pure lines, hybrids, and clonal varieties. The hybrid breeding functionality of ISB was used in Case study IV, i.e. ISB-B4H. Eight output files are generated after running. Output with extension name *.cro contains the number of retained crosses after each cycle of selection. Output with extension name *.gbe contains the genetic variance, environmental variance, genotype by environment interaction variance of traits after each breeding cycle. Output with extension name *.h2b contains the genetic variance and heritability in the broad sense of each trait in each environment. Output with extension name *.res contains the number of plants and families after each generation of selection. Output with extension name *.tgv contains the population mean of genotypic values of pure lines. Output with extension name *.tst contains the population mean of genotypic values of test crosses. Output with extension name *.hbd contains the population mean of genotypic values of crosses with the other heterotic group. Output with extension name *.pox contains the female and male parents in making all crosses at the beginning of each breeding cycle.

1. **Running time and other instructions to run the examples**

It takes about 0.5, 5 and 112 minutes, respectively, to run Drift.exe, Prs.exe and ISB.exe on one example data set in a normal desktop computer.

To run Drift.exe, three input files are needed, which are named by “Drift.gmd”, “Drift.pop” and “Drift.par”. “Drift.gmd” defines the genetic model, “Drift.pop” defines the initial population, and “Drift.par” defines the parameters such as population size, number of cycles, and number of runs.

To run PRS.exe, three input files are needed, which are named by “PRS.gmd”, “PRS.pop” and “PRS.par”. “PRS.gmd” defines the genetic model, “PRS.pop” defines the initial population, and “PRS.par” defines the parameters such as population size, size of family, number of selected individuals, number of cycles, and number of runs.

To run ISB.exe, five input files are needed, which are indicated in the file named “ISB.mio”. In this file, the 1st line (excluding notes starting with “!”) is given by “B4H”, representing one functionality of ISB. The 2nd line specifies a random seed (integer; >0 for fixed seed; <=0 for unfixed seed) in simulation. The 3rd line specifies name of the input file to define Gmodel. The 4th line specifies name of the input file to define one parental or starting population. The 5th line specifies name of the input file to define one tester population. The 6th line specifies name of the input file to define one complimentary heterotic population. The 7th line specifies name of the input file to define breeding methods for hybrid development. The 8th line speficies name of the prefix for output files.

To repeat the case studies on a number of data sets, a batch-command file called “ISB.bat” is provided for Case study IV. “ISB.bat” is in a pure-text format, the users may check its contents. By double click “ISB.bat”, ISB.exe will run sequentially on the four data sets as given in Case study IV.

Input and output files for Case studies I and II are included in folder “Drift with output files”. Input and output files for Case study III are included in folder “PRS with output files”. Input and output files for Case study IV are included in folder “ISB with output files”.
